# Ultrasound on Erect Penis Improves Plaque Identification in Patients With Peyronie’s Disease

**DOI:** 10.3389/fphar.2019.00312

**Published:** 2019-04-04

**Authors:** Yu Liu, Dequan Zheng, Xiaolin Liu, Xiaohong Shi, Shengchun Shu, Jinbing Li

**Affiliations:** ^1^Department of Ultrasonography, Second Clinical Medical College, Guangzhou University of Traditional Chinese Medicine, Guangzhou, China; ^2^Andrology Center, Second Clinical Medical College, Guangzhou University of Traditional Chinese Medicine, Guangzhou, China

**Keywords:** Peyronie’s disease, ultrasound, penis, palpation, lesion identification

## Abstract

**Objectives:**

To compare the sensitivity of identification of penile plaques in the erect and flaccid penises by ultrasound in patients with Peyronie’s disease (PD).

**Materials and Methods:**

A total of 75 PD patients were screened by palpation and ultrasonography for penile lesions in both flaccid and erect penises induced by prostaglandin E1 (PG-1) injection.

**Results:**

A total of 138 lesions were identified by ultrasound in the erect penises induced by injection of PG-1. However, only 74.6% of the lesions (103) were detectable by the palpation of the flaccid penises, and 84.1% (116) by ultrasound of the flaccid penises. The ultrasound confirmed 99 of the palpated lesions in the flaccid penises. The detection rate of lesions in drug-induced erect penises by ultrasound was significantly higher than those in the flaccid penises by the ultrasound (*P* < 0.01) or palpation (*P* < 0.0005) The type of penile lesions identified by ultrasonography included tunical thickening, calcifications, septal fibrosis, and intracavernosal fibrosis. The ratios of these lesions confirmed by ultrasound were 52.6, 33.6, 6.0, and 7.8%, respectively, in the flaccid penises, and 55.8, 28.3, 8.7, and 7.2%, respectively, in the erect penises.

**Conclusion:**

Drug-induced erection can be used in suspicious PD patients when penile lesion is not identified by palpation or ultrasound in the flaccid penis.

## Introduction

Peyronie’s disease (PD) is a common penile disorder with a prevalence between 3.2–8.9% (Schwarzer et al., 2001; Rhoden et al., 2001; Mulhall et al., 2004). PD is caused by the abnormalities in the tunica albuginea and the adjacent tissue of corpora cavernosa of the penis. PD can cause pain, deformity, shorting, as well as bending of the penis during erection, and ultimately results in erectile dysfunction (ED) (Hauck and Weidner, 2001; Ralph et al., 2010). It has been reported that PD is related to the penile plaques formed by aberrant wound healing after minor trauma on the penis (Zargooshi, 2004; Gur et al., 2011). Some PD patients have a genetic predisposition to localized fibrosis formation in response to trauma on the penis (Hauck et al., 2004; Domes et al., 2007).

Peyronie’s disease can be initially diagnosed based on patient’s history and palpation of the penis (Weidner et al., 1997; Hauck et al., 2003), and is subsequently confirmed with evidence of lesions by ultrasound. Ultrasound is able to distinguish calcified tissue from soft tissue in the penis of PD patients. Furthermore, it is more sensitive to find smaller and non-palpable tissue in the penis than palpation alone, and be able to further evaluate the extent of tissue fibrosis (Kalokairinou et al., 2012). The sensitivity to detect a lesion in the penis by ultrasound varies between 39 and 100% (Balconi et al., 1988; Princivalle et al., 1989; Lopez and Jarow, 1991; Vosshenrich et al., 1995; Nicolai et al., 1996; Muralidhar et al., 1996).

Ultrasound is typically conducted on the flaccid penis. However, ultrasound on flaccid penis fails to reveal the lesions which can be detected by palpation in some PD patients, and pharmaceutical induced erection is required (Prando, 2009). Erection stretches the tunica albuginea, and subsequently facilitates the detection of minor lesions by the ultrasound (Kalokairinou et al., 2012). Hypoechoic and isoechoic lesions can be detected with distension or retraction of the corpora cavernosa (Kalokairinou et al., 2012). Although ultrasound has such advantages in diagnosis of PD, it suffers from a systematic comparing the sensitivity in order to find a lesion in flaccid and drug-induced erect penises in PD patients. Therefore, the present study used ultrasound to find penile lesions in a group of Chinese patients with PD. The penis was first examined in the flaccid state followed by a drug-induced erect state.

## Materials and Methods

### Study Patients

All patients with PD were recruited from the Andrology Center of our hospital between the period of March, 2015 to May, 2018. A patient was included in the study if a palpable nodule, focal hardening, or bending of the axis of the penis during erection was found in the flaccid or erect penis. Exclusion criteria were congenital penile curvature and previous PD surgery. A total of 75 patients with median age of 45 years, ranging from 29 to 70 years were eventually included in this study. All participants provided written informed content as well. The study was approved by the Ethics Committee of our institute.

### Penile Examination on PD Patients

A veteran urologist examined the penis on all the PD patients. A palpable penile plaque identified in the flaccid state was further confirmed by stretching the penis with one hand while gently compressing the penile shaft between the fingers and thumb of the other hand. The number, size, and location of the plaques were recorded, and the symptoms of pain were thoroughly evaluated.

### Ultrasound

A Doppler ultrasound was used to visualize penile plaques following palpation. The GE Logq E9 or Philips IU22 units with a 5.0–15.0 MHZ linear-array transducer was used.

Two ultrasound protocols were adopted to compare their sensitivities in diagnosing PD. In the first protocol, the penis was checked at the flaccid state. In the second protocol, 10 mg of prostaglandin E1 (PG-1) was injected into the corpus cavernosum to induce erection. A second dose of PG-1 was administered if the penis was not fully erect. All 6 patients obtained satisfactory erection after the second injection of PG-1. Then, patients were examined in a supine position in a warm and quiet private room. The flaccid and erect penises were longitudinally and transversely checked from the sulcus coronarius to the base. The location, size, number, and morphological characteristics of plaques were recorded. The plaques were further classified into 4 types according to their locations and calcification status; tunical thickening (tunica thickness is greater than 2 mm), septal fibrosis, intracavernous fibrosis, and penile calcifications (Smith et al., 2009; Breyer et al., 2010; Chung et al., 2011, 2012).

### Statistical Analysis

All data were statistically analyzed with SPSS 12.0 software (SPSS Inc., Chicago, IL, United States). The number and types of plaques were analyzed with the Wilcoxon test. A *P*-value <0.05 was defined as statistically significant.

## Results

### Patients’ Demographic Information and Clinical Complaints

Patients had various symptoms or noticed penile abnormalities for a duration of 4 to 42 months (Table 1). Pain during intercourse was the predominant complain in PD patients (49.33%). The penile abnormalities found in PD patients included curvature in erect state (26.67%), decreased penile rigidity (38.67%), penis shortening (9.33%), and inability to sustain erection (8.00%). Approximately 11% of patients reported a history of penile bruising or significant penile pain after intercourse.

**Table 1:** Patients’ demographic information.

	Number (*n* = 75)
Age (years)	45 (29–70)
Duration of symptoms (months)	11 (4–41)
Penile trauma (%)	8(10.66)
Penile pain (%)	37 (49.33)
Penile curvature (%)	20 (26.67)
Penile shortening (%)	7 (9.33)
Decreased penile rigidity (%)	29 (38.67)

### Sonographic Characteristics of Plaques in Erect Penis

A total of 138 penis plaques were identified by ultrasound in 75 PD patients after PG-1 induced erection. Approximately 52% of the plaques were less than 1.5 cm in length, 37% ranged from 1.5 to 3.0 cm, and 11% were larger than 3.0 cm. The thickness of the plaques varied from 0.2 to 1.6 cm.

Ultrasound was able to distinguish penile plaques with different characteristics. Approximately 28.3% of the plaques were calcified, in which 15.9% were fibrosis, and 55.8% were tunical thickening (Table 2). Among the 99 non-calcified plaques, 10(10.1%) showed hypoechoic lesions, 21(21.2%) showed isoechoic lesions and 68(68.7%) showed hyperechoic lesions.

**Table 2:** Types and percentages of penile plaques identified by ultrasound in PD patients (*n* = 75).

Group	US_F_	US_E_
Tunical thickening	61 (52.6%)	77 (55.8%)
Calcification	39 (33.6%)	39 (28.3%)
Septal fibrosis	7 (6.0%)	12 (8.7%)
Intracavernosal fibrosis	9 (7.8%)	10 (7.2%)
Total	116	138^∗^

*US_F_, ultrasound of the flaccid penis; US_E_, ultrasound of the erect penis. ^∗^Number of lesions found by US_E_ was significantly higher than US_F_, p < 0.005.*

### Plaque Was Easily Identified in Erected Penis

**FIGURE 1:**
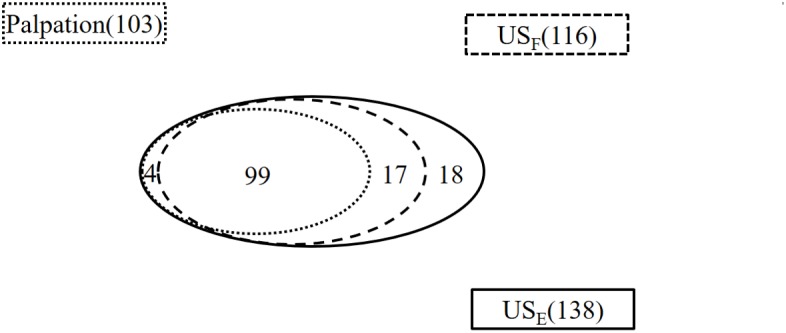
The number of plaques found by palpation and ultrasound in the flaccid and erect penis. US_F_, ultrasound of the flaccid penis; US_E_, ultrasound of the erect penis.

Compared with the number of penis plaques (138) found in the erect state, palpation in the flaccid state alone was able to identify 74.6% of them (103/138) in 71 out of 75 patients (Figure 1). The other 4 patients failed to display any focal or diffuse alteration in the penis by palpation, though they felt vague pain in the ventral base of the erect penis. All of the plaques were painless when the penis was in the flaccid state. The majority of plaques (90.29%) were palpable on the dorsal surface of the penis, followed by the ventral surface (7.77%), and the lateral surface (1.94%).

**FIGURE 2:**
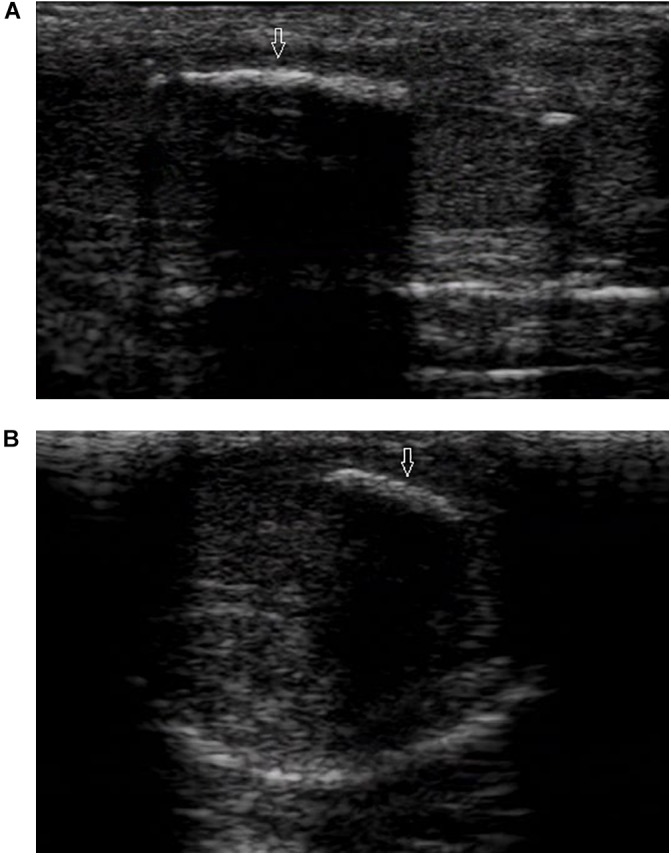
A large calcified lesion in the tunica albuginea of the flaccid penis. **(A)** Longitudinal sonogram, dorsal access. Large calcified plaques (arrow) in the dorsal side with strong shadow caused dorsal curvature of the penis. **(B)** Axial sonogram, dorsal access. Plaque located in the tunica albuginea of left corpora cavernosa.

**FIGURE 3:**
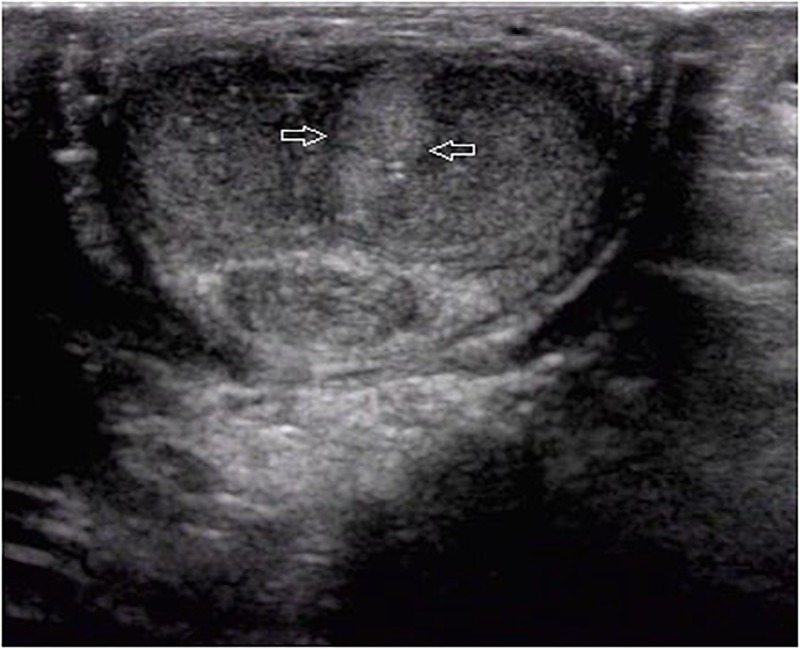
A septal fibrosis in the flaccid penis. Axial sonogram, ventral access, the hyperechoic fibrosis (arrows) in the septum.

Ultrasound detected 84.1% (116/138) of the plaques in 71 out of 75 patients when the penis was examined in the flaccid state. The frequencies of tunical thickening, calcifications (Figure 2), septal fibrosis (Figure 3), and intracavernosal fibrosis were 52.6, 33.6, 6.0, and 7.8%, respectively. The sonographic examination did not reveal any type of focal or diffuse alteration of penile tissue in 4 patients in the flaccid state, though they had a small dorsal nodule palpable.

### Numbers of Identified Plaques Were Significantly Different Among Three Examination Techniques

**FIGURE 4:**
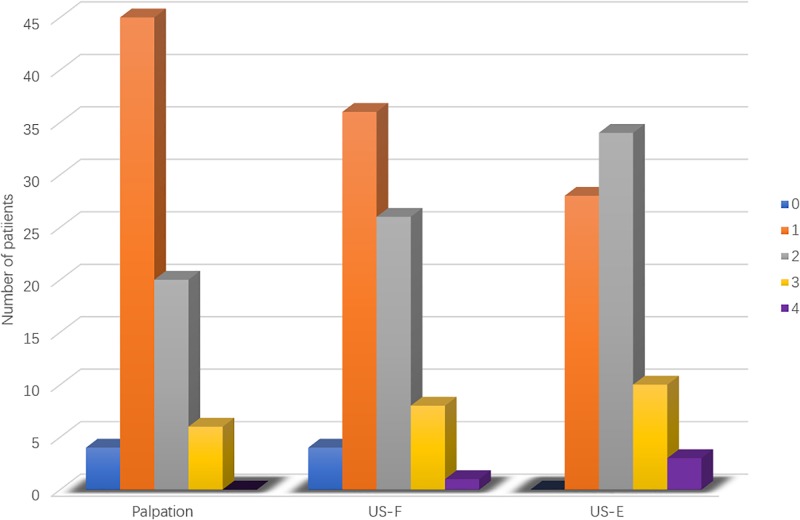
Frequency of penile plaques in PD patients. Comparisons of palpation, ultrasound in the flaccid penis (US-F), and ultrasound in the erect penis (US-E). 0 to 4, number of lesions.

Palpation of the flaccid penis alone found one single plaque in each of 45 patients, 2 isolated plaques in 20 patients, and 3 plaques in the other 6 patients (Figure 4). Ultrasound of flaccid penis identified 36 patients with solitary plaque, 26 patients with 2 isolated plaques, 8 patients with 3 lesions, and 1 patient with 4 lesions. These findings were significant different (*P* < 0.05) in comparison with palpation. Application of ultrasound on drug-induced erection revealed that 28 patients had solitary lesion, 34 had 2 separate lesions, 10 had 3 lesions, and 3 had 4 lesions, which was significantly different from those with palpation (*P* < 0.0005) or ultrasound in the flaccid penis (*P* < 0.01).

### Sonography Identified Plagues More Efficiently in Erect Penis in PD Patients

Ultrasound detected 99 of the 103 palpable lesions (96%) in flaccid penis. However, 17 additional lesions were found by ultrasound imaging. In addition, 8 out of the 17 additional lesions were located at the intracavernosum, 6 at the septum, and 3 at the ventral tunica albuginea of corpora cavernosa behind the corpus spongiosum. Therefore, a total of 116 plaques were identified in the flaccid penis with ultrasound.

**FIGURE 5:**
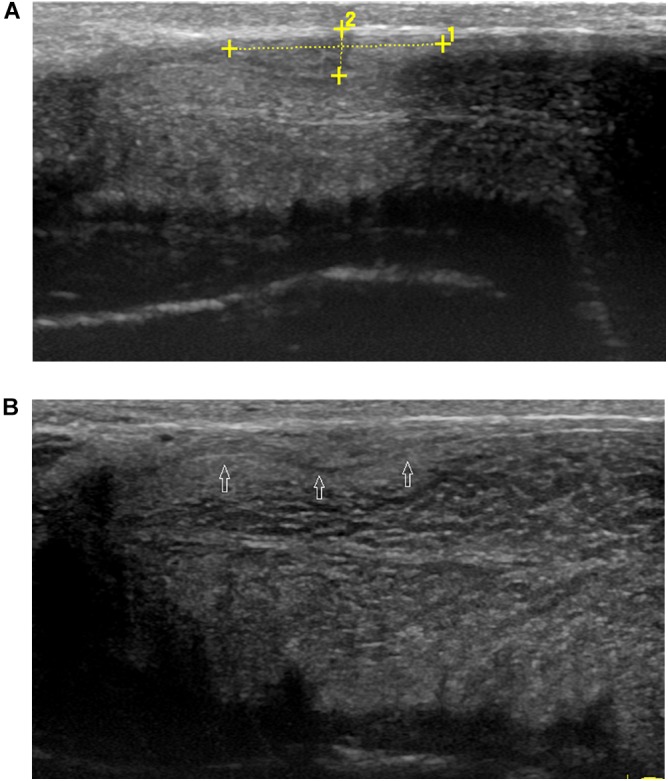
Palpable ventral plaque of left corpus cavernosum. **(A)** Longitudinal sonogram, oblique ventral access (from the midline to the left lateral face of the penis). Fuzzy ventral tunica thickening of left corpus cavernosum in the flaccid penis. **(B)** Longitudinal sonogram, oblique ventral access (from the midline to the left lateral face of the penis). With the corpora cavernosa expansion, the local tunica albuginea thickening becomes obvious (arrows) in the erect penis.

**FIGURE 6:**
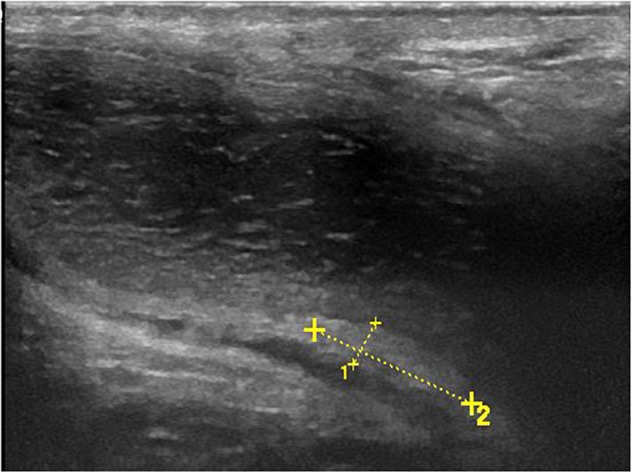
Longitudinal sonogram (ventral access) of a palpable dorsal nodule. This nodule was not detected by the sonography in the flaccid penis. The sonography in erect penis shows obvious dorsal tunica albuginea thickening at the root of penis.

**FIGURE 7:**
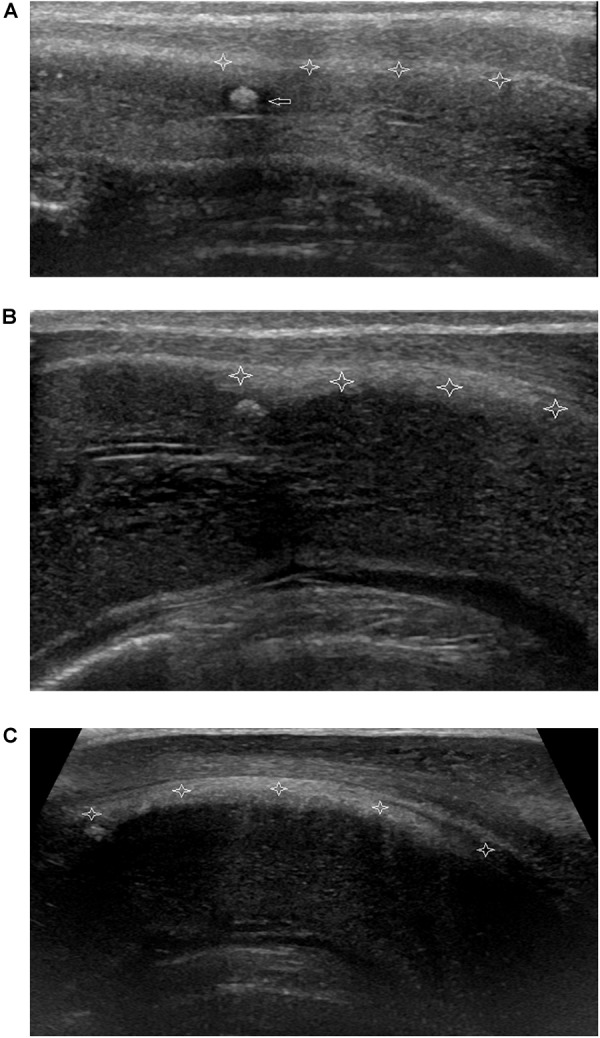
Non-palpable nodule of focal thickening of the ventral tunica albuginea of corpora cavernosum behind corpora spongiosum at the root of penis. **(A)** Longitudinal sonogram, ventral access. No focal thickening of tunica albuginea of corpora cavernosum (stars) is shown and a hyperechoic fibrous plaque (arrow) in the corpora cavernosum in flaccid penis. **(B)** Longitudinal sonogram, ventral access. With the corpora cavernosa expansion, the local tunica albuginea shows thickening (stars) with the weak acoustic attenuation in the erect penis. **(C)** Longitudinal sonogram, ventral access. The acoustic attenuation behind the thickening tunica albuginea becomes strong when the penis reaches complete erection.

All 120 lesions detected with palpation or sonography in the flaccid penis were identified in the erect penis by ultrasound (Figure 5). The plaques undetectable in the 4 patients by ultrasound in the flaccid penis were confirmed to be tunica albuginea thickening at the root of penis by the ultrasound in erect penis (Figure 6). One of four patients who complained of vague pain in the ventral side of the base of erect penis failed to palpate any focal lesion, however, a large ventral tunica thickening was found by ultrasound with strong acoustic attenuation at the root of erect penis. No focal thickening of tunica albuginea was detected in the flaccid penis by ultrasound in this patient (Figure 7). In addition, 17 additional lesions were detected by ultrasound during erection including 11 tunical thickenings, 5 fibrosis at the septum, and 1 fibrosis in the intracavernosum. In comparison with ultrasound in the flaccid penis, the number of plaques found by the ultrasound in the erect penis was significantly higher than those in the former (*P* < 0.01).

## Discussion

The present study, to the best of knowledge, for the first time investigated the sensitivity of ultrasound in identifying penile plaques in Chinese PD patients. More penile plaques were identified when ultrasound was performed on a prostaglandin-induced erect penis compared with a flaccid one. The findings provide evidence that drug-induced erection modality can be used in patients with suspicious PD history and palpation but negative ultrasound findings in the flaccid penis.

PD may be diagnosed by palpation of penile plaques (Hauck and Weidner, 2001). However, palpation alone is difficult to find lesions in the septum, the intracavernosum, and the ventral tunica albuginea of the corpora cavernosa behind the corpus spongiosum due to their anatomic locations. Therefore, penile ultrasound is commonly used to confirm the diagnosis, and some penile lesions can only be identified by the ultrasound, e.g., the septal fibrosis, intracavernosal fibrosis, or sub-tunical calcifications (Brant et al., 2007). The present study confirmed the ability of ultrasonography to identify more penile plaques than palpation in PD patients. Similar findings were reported by Prando et al. that the ultrasound screening found penile plaques in 28 of 78 PD patients (35.8%) who had not reported any penile lesion by palpation alone (Prando, 2009). Therefore, penile ultrasound screening is a valuable tool to find lesions in patients with PD, especially lesions in the intracavernosum and septum.

In the present study, the ultrasound was able to detect 96 and 100% of palpated penile plaques in the flaccid and erection penis, respectively. Our results are similar to some studies (Balconi et al., 1988; Princivalle et al., 1989; Prando, 2009), however, they showed a higher detection rate than other studies, which ranged from 39 to 72% (Lopez and Jarow, 1991; Vosshenrich et al., 1995; Nicolai et al., 1996; Hauck et al., 2003). The discrepancy between the present study and the others may be due to different ultrasound equipment used, different frequencies of the probes, different ethnic group of PD patients, and different experience of sonographers.

The present study demonstrated that the ultrasound performed better in identifying penile lesions when the penis was in the erect state. Dorsal palpable penile nodules in 4 PD patients were not detected by the ultrasound in the flaccid penis; however, they were well characterized by ultrasound in the erect penis. Furthermore, 18 new lesions were discovered by the ultrasound in the erect penis. The tunica albuginea in the flaccid penis is thick, which can attenuate signal from the lesions within the corpora cavernosa. However, the tunica albuginea is stretched and becomes thinner due to the expansion of the corpora cavernosa in the erect penis. A penile lesion, which is usually not stretchable, can then be visualized by ultrasound due to the increased contrast enhancement between the lesion and peripheral normal tissue. Therefore, ultrasound in an erect penis can increase the chance of identifying a lesion which is not well contrast with the surrounding tissues, especially in the tunica albuginea and septum. However, the ultrasound was able to identify calcified lesions efficiently regardless of the penis state, which may be due to the distinctive shadow generated by the calcified lesions.

The present study had some limitations. Given the size of our patient population, and not all types of PD plaques were represented. In addition, ultrasound images were provided by one sonographer in both the flaccid and erect penis, which may cause bias in the data collection. Therefore, a second independent sonographer should be included in the future study.

## Conclusion

The ultrasound screening of penile plaques in the erect penis was more sensitive than in the flaccid state for Chinese PD patients. Ultrasound of the erect penis can improve identification of lesions which are not obvious, especially lesions in the tunica albuginea and septum. Our results also suggest that ultrasound in erect penis should be considered in all suspicious PD patients in the clinical routine.

## Data Availability

All datasets generated for this study are included in the manuscript and/or the supplementary files.

## Author Contributions

JL, YL, and DZ conceived of the study, participated in its design and drafted the manuscript. JL performed the examination of ultrasonography. XL and XS helped in data acquisition, analysis and interpretation. XL and SS helped in literature review and statistical analysis.

## Conflict of Interest Statement

The authors declare that the research was conducted in the absence of any commercial or financial relationships that could be construed as a potential conflict of interest.
